# *Campylobacter jejuni*/*coli* Infection: Is It Still a Concern?

**DOI:** 10.3390/microorganisms12122669

**Published:** 2024-12-23

**Authors:** Piero Veronese, Icilio Dodi

**Affiliations:** Pediatric Infectious Disease Unit, Barilla Children’s Hospital of Parma, 43126 Parma, Italy; idodi@ao.pr.it

**Keywords:** *Campylobacter*, foodborne illness, antibiotic resistance, pediatric population

## Abstract

Campylobacteriosis is a leading cause of infectious diarrhea and foodborne illness worldwide. *Campylobacter* infection is primarily transmitted through the consumption of contaminated food, especially uncooked meat, or untreated water; contact with infected animals or contaminated environments; poultry is the primary reservoir and source of human transmission. The clinical spectrum of *Campylobacter jejuni*/*coli* infection can be classified into two distinct categories: gastrointestinal and extraintestinal manifestations. Late complications are reactive arthritis, Guillain–Barré syndrome, and Miller Fisher syndrome. In the pediatric population, the 0–4 age group has the highest incidence of campylobacteriosis. Regarding the use of specific antimicrobial therapy, international guidelines agree in recommending it for severe intestinal infections. Host factors, including malnutrition, immunodeficiency, and malignancy, can also influence the decision to treat. The Centers for Disease Control and Prevention (CDC) has identified antibiotic resistance in *Campylobacter* as a ‘significant public health threat’ due to increasing resistance to FQs or macrolides. Although numerous vaccines have been proposed in recent years to reduce the intestinal colonization of poultry, none have shown sufficient efficacy to provide a definitive solution.

## 1. Introduction

*Campylobacter jejuni* and *coli* are the etiological agents of campylobacteriosis, one of the most prevalent gastroenteritis worldwide. This foodborne infection is primarily due to the ingestion of raw or undercooked meat or untreated water. Intestinal infection is usually self-limiting and in most cases presents with mild symptoms [1,2]. More severe forms can be observed in children, infants, or immunocompromised individuals. Extraintestinal localizations are possible, albeit rare. Of significant clinical importance are late complications, such as Guillain–Barré syndrome and reactive arthritis, conditions in which a previous *Campylobacter* infection plays a primary role [3]. There has been an alarming escalation of multidrug resistance in poultry populations over the past few decades. Effective control of *Campylobacter* requires a multi-step approach with pre- and post-harvest interventions, including adherence to and improvement of biosafety and hygiene standards, employment of specific food additives, and the development of an effective vaccine [4].

## 2. Microbiology

*Campylobacters* are Gram-negative, curved, or spiral- shaped motile, non-spore-forming, and microaerophilic bacilli. They have an optimal growth at temperatures ranging from 37 °C to 42 °C [5].

The genus *Campylobacter* is now known to consist of 57 species, which can be classified into 5 phylogenetic groups. A taxonomic listing of all currently recognized *Campylobacter* species is presented in Table 1 [6,7,8]. However, *Campylobacter jejuni* is currently the most clinically significant species of the genus. *Campylobacter jejuni* can be further subdivided into two subspecies: *Campylobacter jejuni subspecies jejuni* and *Campylobacter jejuni subspecies doylei*. In humans, they are responsible for acute febrile enteritis. Another highly prevalent species is *Campylobacter coli*, accounting for up to 25% of human *Campylobacter*-associated infections [6].

Other emerging species include *C. sputorum*, *C. upsaliensis*, *C. ureolyticus*, *C. lari*, and *C. hyointestinalis* [9]. These microorganisms are commonly found as commensals in the gastrointestinal tracts of various animals, such as birds, poultry, dogs, and livestock [10,11]. Human infection is typically acquired via the fecal–oral route through contaminated food, water, or milk, with zoonotic transmission from direct animal contact being less common [12,13].

**Table 1:** Summary of *Campylobacter* phylogenetic groups and related hosts.

Phylogenetic Group	*Campylobacter* Species	Reported Host
*C. jejuni* group	*C. jejuni* subsp. *jejuni*	poultry [14], cattle [15]
	*C. jejuni* subsp. *doylei*	human [16]
	*C. coli*	human [17], poultry [17], cattle [18], dog [19], pig [20], sheep [18]
	*C. hepaticus*	poultry [21]
	*C. helveticus*	human, dog, cat [22]
	*C. upsaliensis*	human, dog, cat [23]
	*C. cuniculorum*	rabbit [24]
	*C. avium*	poultry [25]
	*C. troglodytis*	human, monkey [26,27]
	*C. canadensis*	birds [7]
*C. lari* group	*C. lari* subsp. *lari*	shellfish [28]
	*C. lari* subsp. *concheus*	
	*C. insulaenigrae*	human [29], pinnipeds [30]
	*C. volucris*	human [31], gull [32]
	*C. peloridis*	shellfish [28]
	*C. subantarticus*	birds [33]
	*C. ornithocola*	birds [34]
*C. concisus* group	*C. concisus*	human [35], dog, cat [36]
	*C. showae*	human, dog [37]
	*C. rectus*	human, dog [38]
	*C. curvus*	
	*C. mucosalis*	
	*C. pinnipediorum*	pinnipeds [39]
*C. ureolyticus* group	*C. ureolyticus*	human [40], cattle [41]
	*C. hominis*	human [42]
	*C. geochelonis*	tortoise [43]
	*C. corcagienesis*	monkey [44]
	*C. gracilis*	human [45], dog [38]
	*C. sputorum*	human [46], cattle [47], dog [38]
*C. fetus* group	*C. fetus subsp. fetus*	human, cattle [48]
	*C. fetus subsp. venerealis*	
	*C. fetus subsp. testudinum*	
	*C. hyointestinalis*	human, cattle, dog, hamster [49]
	*C. iguaniorum*	reptiles [50]
	*C. lanienae*	human [51], cattle, pig [52], chinchilla [53]

## 3. Epidemiology

Campylobacteriosis, caused by *Campylobacter jejuni* or *coli* infection, is a leading cause of infectious diarrhea worldwide. Despite the limitations imposed by inadequate national surveillance in numerous African, Asian, and Middle Eastern countries, the incidence of infection appears to be increasing in recent decades. According to the Centers for Disease Control and Prevention (CDC), the annual burden of infection in the United States is substantial, with an estimated 1.5 million cases and a total economic impact ranging from 1.3 to 6.8 billion dollars [40].

In Europe, *Campylobacter* is the most common cause of bacterial enteritis, the leading cause of foodborne illness, and accounts for over 60% of all reported zoonoses [54]. National surveillance in Asia is limited to Japan, Korea, and Singapore; epidemiological data from these countries are consistent with those observed in other high-income nations. Campylobacteriosis is the leading cause of bacterial enteritis in both Australia and New Zealand [55].

Africa is estimated to have the world’s highest incidence of campylobacteriosis [56]. Despite the challenges in diagnosis and data collection in low-resource settings, the number of infections is estimated to be steadily increasing, particularly among children. This trend may be linked to the growing consumption of animal protein, notably poultry, in these regions [57]. There is growing recognition of the association between *Campylobacter* spp. and other conditions, such as stunting [58].

Bimodal incidence patterns are characteristic of this infection: the first and larger peak occurs in preschool-aged children, under 5 years old; the second peak occurs in young adults [59,60]. In Europe, infections in children under 5 years of age account for approximately 13% of all reported cases. In temperate regions, there is an increase in cases during the warmer months. The seasonal pattern is strongly suggestive of a causal relationship with increased consumption of undercooked foods and compromised cold chain integrity. Nevertheless, host gene expression may play a role in this marked seasonal pattern [61].

## 4. History

Theodor Escherich provided the earliest credible account of *Campylobacter* when, in 1886, he observed spiral-shaped bacteria in a biological specimen and termed them ‘cholera infantum’ [62].

Following these findings, veterinarians McFadyean, Stockman, and Smith isolated and identified germs, described as ‘vibrios’ and ‘spirillum’, as the cause of abortion in sheep and cattle, respectively, in 1909 and 1919, naming them *Vibrio fetus* [63,64]. Subsequent investigations by Jones et al. in 1931 and Doyle in 1944 implicated *Vibrio jejuni* as the causative agent of dysentery in calves and swines, respectively [65]. Until the 1950s, these microorganisms were exclusively linked to veterinary diseases, causing septic abortion in cattle and sheep, as well as enteric diseases in livestock [66]. Indeed, it was not until 1957 that King isolated bacteria from human blood cultures, which he termed ‘related vibrio’, due to their similarity to *Vibrio fetus*, first isolated by Vinzent in 1947 [67,68]. Since 1968, the work of Butzler and Dekeyser has been pivotal in highlighting the pathogenic role of these bacteria [69,70]. Moreover, their work provided the initial evidence of the susceptibility of these bacteria to erythromycin treatment [71].

The substantial progress made in diagnostic techniques, especially since the 1970s, has significantly contributed to our expanding knowledge of this pathogen. Since the 1970s, the first reports of *Campylobacter* colitis emerged from numerous tropical, European, and North American regions. Notably, recent advances in molecular biology have shed light on the molecular mechanisms governing bacterial persistence and virulence.

## 5. Transmission, Environmental Reservoirs, and Risk Factors

*Campylobacter* infection is primarily transmitted through the consumption of contaminated food or contact with infected animals or contaminated environments. Poultry is the primary reservoir and source of human transmission; campylobacteriosis is therefore considered a zoonosis. The predominant risk factors involve the consumption of contaminated food products, including raw or undercooked meat, milk, vegetables, and water [72].

In high-resource countries, infections are typically sporadic cases, frequently associated with contaminated food consumption or returning from travel to endemic areas. In contrast, in low-resource settings, campylobacteriosis is often endemic, with little or no seasonal variation [73,74].

In these settings, consumption of contaminated water or unpasteurized milk is undoubtedly the primary route of transmission; however, the increased consumption and, consequently, exposure to meat should also be highlighted [75].

Immunodeficiency represents a substantial host risk factor for campylobacteriosis; according to Kownhar et al., patients with HIV have higher rates of campylobacteriosis compared to healthy individuals [76].

Domestic animals are the primary environmental reservoirs that should be considered. Poultry is undoubtedly a major carrier; however, the environments surrounding farms can also be colonized by the germ, from soil to water to building surfaces [77]. Moreover, these animal species, as well as other avian species like ducks, are reservoirs for other *Campylobacter* species, notably *C. lari*, *upsaliensis*, and *concisus* [78]. Contamination of meat products extends to other animal species, including swine and cattle, although ovine meat is less commonly implicated [15,79,80]. As highlighted by Moore et al. fresh and frozen meats exhibit a higher prevalence of contamination compared to pre-cooked products [66]. It is well-established that wild animals, especially avian species, can serve as reservoirs for the bacteria. Human-to-human transmission is possible, although less significant.

**Figure 1:**
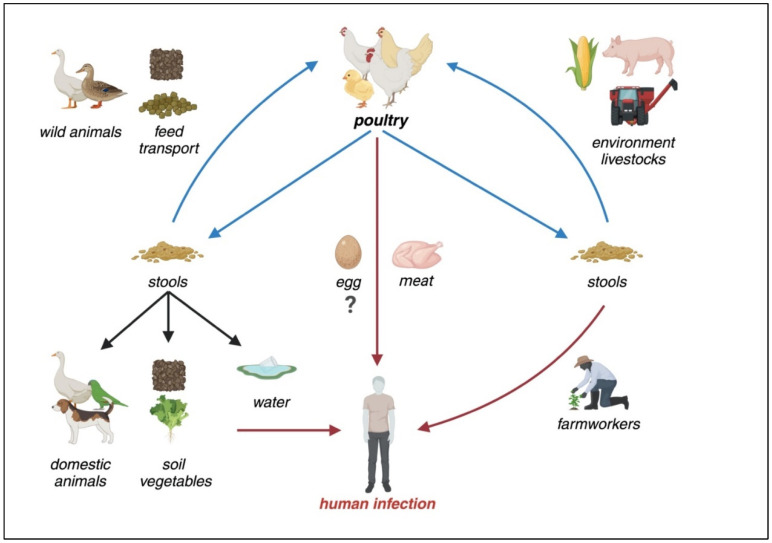
Transmission, environmental reservoirs, and risk factors for human *Campylobacteriosis*. As a zoonotic disease, poultry is the primary reservoir for *Campylobacter*. In non-endemic regions, consuming raw or undercooked poultry meat and direct contact with animals are the primary risk factors. However, in endemic areas, environmental contamination, including water sources, along with poultry contact, increased meat consumption, and inadequate hygienic practices contribute to the widespread persistence of the bacterium.

Figure 1 provides a schematic overview of the mechanisms by which *Campylobacter* persists in environmental reservoirs and is transmitted to humans. Poultry plays a central role in this process, with the persistence of the bacterium in poultry meat, combined with factors such as transportation and environmental contamination, facilitating transmission.

## 6. Virulence Factors and Pathogenesis

Key virulence factors of *Campylobacter* mediate motility, chemotaxis, adhesion, translocation, and invasion; furthermore, there are pro-inflammatory effector molecules and the *Campylobacter*’s ability to form biofilms.

Motility is conferred by the flagellar apparatus, and chemotaxis primarily by transducer-like proteins (Tlps). Adhesins mediate adhesion, while secretion systems such as the Type III secretion system (T3SS) and the Type VI secretion system (T6SS), along with *Campylobacter* invasion antigens (Cia) and high-temperature requirement protein A (HtrA), enable bacterial invasion of the host mucosa. Finally, pro-inflammatory factors and biofilm formation are involved in both *Campylobacter* virulence and persistence.

Motility plays a crucial role in the invasion of the intestinal mucosa of the jejunum, ileum, and colon, enabling the bacterium to reach the epithelial cells by traversing the viscous mucus layer. Flagellar motility at both poles, coupled with the spiral morphology, is responsible for bacterial movement [81]. *Campylobacter* coordinates its two opposing flagella when swimming in viscous environments. Additionally, these organelles allow the pathogen to overcome certain host defense mechanisms, such as peristalsis. The flagellar apparatus confers multiple capabilities to *Campylobacter*: Song et al. demonstrated that the flagellum can also evade TLR5 activation and subsequent immune response [82]. Additionally, it promotes adhesion to and invasion of intestinal epithelial cells (IECs) as well as biofilm formation. This is achieved through the secretion of Cia via the Type III secretion system (T3SS), the most complex and widespread secretion system among Gram-negative bacteria [83,84,85]. Cia proteins serve as effector molecules, and their production, and secretion are regulated by environmental factors and host-derived products. In fact, their secretion is triggered upon contact with IECs and is likely modulated by host cell-derived factors [86]. Specifically, CiaB, CiaC, and CiaD appear to facilitate bacterial uptake by host cells. CiaD, as shown by Samuelson et al., also promotes the secretion of pro-inflammatory cytokines, such as interleukin 8 (IL-8), and facilitates epithelial invasion [87]. CiaI protein seems to be essential not only for invasion but also for bacterial persistence at mucosal sites [88]. Indeed, as reported by Watson and Galán, *Campylobacter* is able to survive within IECs as a facultative intracellular pathogen thanks to the so-called *Campylobacter*-containing vacuoles (CCVs) [89]. CiaI protein prevents the fusion of CCVs with host cell lysosomes, promoting intracellular persistence [84].

Despite the central role of T3SS, the *Campylobacter* secretion system likely involves other molecules such as the Type IV secretion system (T4SS) and the Type VI secretion system (T6SS). T4SS is a widespread secretion system found in both Gram-positive and Gram-negative bacteria, such as *Helicobacter pylori* [90]. This system mediates the translocation of effector molecules through the bacterial cell envelope and into the host cell. T4SS-encoding genes are located in plasmids or genomic pathogenicity islands (PAIs), facilitating their horizontal transfer among different bacterial species [91]. Despite observations by Bacon et al. suggesting a potential role for T4SS, its actual presence in *Campylobacter* remains unclear [92,93]. T6SS plays a clear role in host invasion. According to Lertpiriyapong et al., T6SS mediates both invasion and adhesion to colon cells. Notably, it is sensitive to deoxycholic acid, favoring colonization of the proximal colon where levels of this bile-salt are lower compared to the small intestine [94]. Moreover, T6SS appears to confer increased oxidative stress resistance to *C. jejuni*, promoting virulence and survival within the host [95]. However, T6SS is present in 16–20% of isolates [94,96].

Recent studies have emphasized the role of HtrA, a serine protease, in bacterial invasion and translocation processes. Once secreted, either freely or within outer membrane vesicles (OMVs), HtrA is capable of disrupting intercellular junctions by targeting E-cadherins. Through this mechanism, *Campylobacter* is able to reach the lamina propria and, subsequently, the systemic circulation [97].

*C. jejuni* capsular polysaccharide (CPS) is a remarkable structure in the outermost surface of the bacterium. It is responsible for both virulence and immune evasion; notably, CPS plays a key role in evading the host complement system [98,99,100,101].

Adhesins, also known as Microbial Surface Components Recognizing Adhesive Matrix Molecule(s) (MSCRAMMs), are present on the outer surface of *Campylobacter*, including the well-characterized *Campylobacter* adhesion to fibronectin (CadF) and Fibronectin-like protein A (FlpA). These molecules are termed fibronectin-binding proteins (FNBPs) due to their ability to bind fibronectin, which is located on the basolateral side of the intestinal epithelium [102]. It is plausible, however, that the multistep invasion process involves a variety of adhesins.

The cytolethal distending toxin (Cj-CDT) is a key virulence factor of *Campylobacter*, and it is also produced by other intestinal pathogens, including genus *Salmonella*, *Escherichia coli* (Ec-CDT), *Shigella dysenteriae* (Sd-CDT), and *Haemophilus ducreyi* (Hd-CDT) [103,104,105,106,107]. Cj-CDT is composed of three subunits: Cj-CDTA, B, and C. The precise mechanisms by which CDT facilitates invasion are not yet fully elucidated; however, its pivotal role in this process is indisputable. It is currently considered a genotoxin; indeed, it can modulate the inflammatory response by inhibiting the phosphatidylinositol-3-kinase (PI-3K) signaling pathway, thereby increasing the production of pro-inflammatory cytokines such as interleukin 1-beta (IL-1beta), tumor necrosis factor-alpha (TNF-alfa), and interleukin-6 (IL-6). Interleukin-8 (IL-8) induction, as demonstrated by Hickey et al., contributes to the intestinal inflammatory state and promotes chemotaxis [108].

The ability to form a biofilm is a crucial factor contributing to the survival and persistence of the germ within the host. Indeed, *Campylobacter* can form both species-specific and polymicrobial biofilms, such as with *Pseudomonas* spp [109]. This occurs through the production of an extracellular polymeric substance (EPS) matrix [83]. Recently, it has been observed that exposure to atmospheric oxygen tension can upregulate biofilm production, enhancing the germ’s survival capacity [110,111].

**Figure 2:**
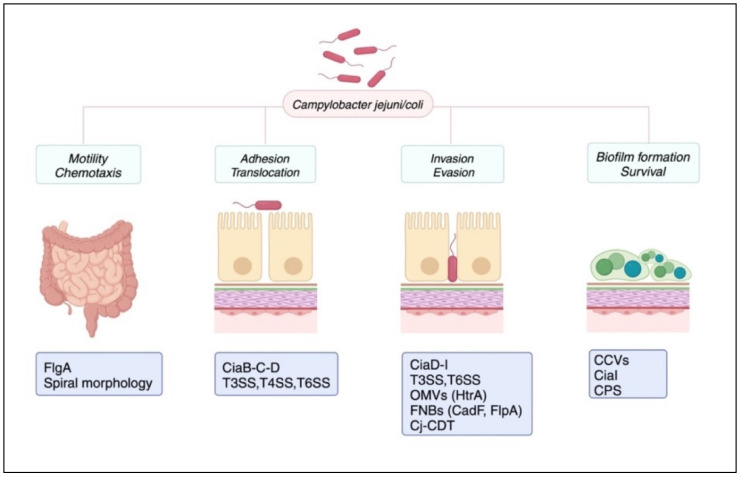
*Campylobacter jejuni/coli* virulence factors. The main pathogenic factors of *Campylobacter* have been categorized into four broad groups in this image: motility and chemotaxis, adhesion and translocation, invasion and evasion of the host immune system, and survival and biofilm formation. The flagellar apparatus, encoded by the *FlgA* gene, is the factor that confers motility to the bacterium, counteracting peristaltic movements. Additionally, it is involved in the secretion of effector molecules (*Campylobacter* invasion antigens, Cia), in the evasion of TLR5-mediated immunity, and in the biofilm formation. CiaB, CiaC, and CiaD facilitate bacterial uptake by and invasion of host cells. The type 3 secretion system (T3SS), a key virulence factor in many Gram-negative pathogens, is primarily responsible for the secretion of Cia effectors. Despite T4SS presence in *Campylobacter* remaining unclear, T6SS plays a clear role in host invasion, mediating both invasion and adhesion to colon cells. During invasion, *Campylobacter jejuni* cytolethal distending toxin (Cj-CDT). Capsular polysaccharide (CPS) and *Campylobacter*-containing vacuoles (CCVs) act as an immune shield, allowing the bacterium to persist within the host.

A schematic representation of virulence determinants of *Campylobacter* is shown in Figure 2.

## 7. Clinical Manifestations

**Figure 3:**
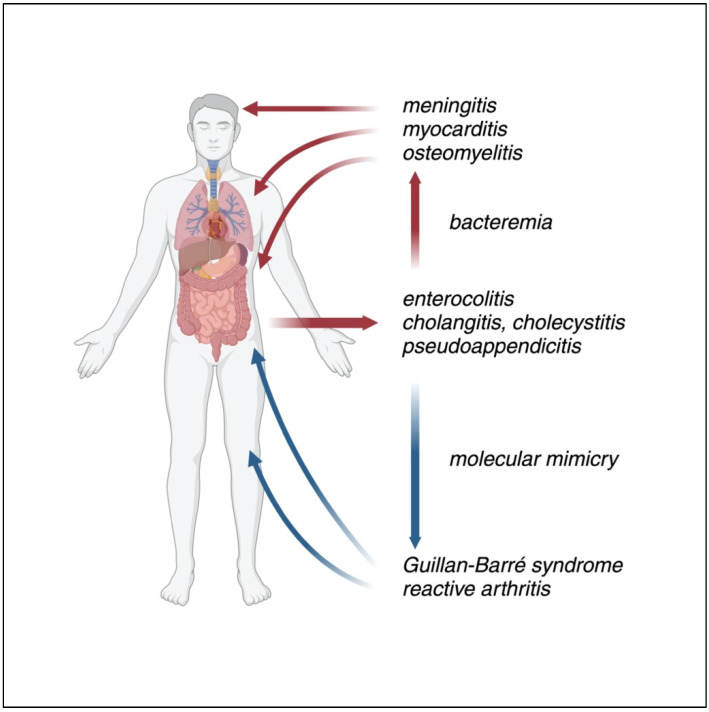
Clinical manifestations and late complications of *Campylobacter* infection. While uncommon, bacteremia is a key factor in the systemic spread of the bacteria following intestinal infection, leading to potential complications in distant organs. Molecular mimicry underlies the pathogenesis of late complications.

The clinical spectrum of *Campylobacter jejuni*/*coli* infection can be classified into two distinct categories: gastrointestinal and extraintestinal manifestations. Gastrointestinal manifestations are by far the most common, including gastroenteritis, colitis, pseudo appendicitis, esophageal and periodontal involvement, cholecystitis, and neoplastic conditions such as cancer and lymphoma. Extraintestinal manifestations, often considered complications, include cutaneous manifestations (such as rash, vasculitis, and erythema nodosum), as well as osteomyelitis, cellulitis, myocarditis, pericarditis, and meningitis. Among the most well-known late complications are reactive arthritis, Guillain–Barré syndrome, and Miller Fisher syndrome [40,112,113]. Figure 3 schematizes the primary clinical manifestations associated with *Campylobacter*.

### 7.1. Gastrointestinal Manifestations

#### 7.1.1. Gastroenteritis

Gastroenteritis and colitis are the predominant clinical manifestations of *Campylobacter jejuni*/*coli* infection. The clinical phenotype mirrors that of other bacterial enteritis, manifesting with fever, acute diarrhea, frequently accompanied by muco-sanguineous stools, and abdominal cramps [6]. Clinically, the presentations of *C. jejuni* and *C. coli* infections are indistinguishable, overlapping significantly with those caused by other bacterial enteropathogens, including *Salmonella* and *Shigella*. The incubation period is usually short, ranging from 1 to 7 days, with an average of about 3 days. An infectious dose as low as 500 pathogens may be sufficient; however, high infectious doses appear to be associated with shorter incubation periods [114,115]. Furthermore, impairment of the gastric acid barrier promotes colonization by the pathogen. The onset of symptoms typically occurs within 72 h of consuming contaminated food, with peak severity lasting 24 to 48 h. The primary site of infection is the jejunum and ileum, with subsequent involvement of the colon. Lamina propria invasion and chemotaxis are the primary pathogenic mechanisms contributing to the muco-hemorrhagic colitis associated with *Campylobacter* [116].

#### 7.1.2. Cholecystitis

*Campylobacter* is a rare infectious agent that can lead to cholecystitis with or without associated diarrhea. The majority of reported cases in the literature describe cholangitis in adults. Pre-existing gallbladder and biliary tract conditions, particularly cholelithiasis and neoplasms, are often identified as predisposing factors in reported cases [117]. Hematogenous spread, cholecystoduodenal reflux, and intestinal bacterial translocation are likely routes of *Campylobacter* dissemination to the biliary tract [118]. Diagnosis is usually presumptive based on stool cultures; however, isolation from bile or tissue specimens, while rare, can confirm the diagnosis.

#### 7.1.3. Pseudoappendicits

Inflammation involving the ileum, ileocecal valve, and cecum can mimic acute appendicitis, particularly when diarrhea is absent. This clinical presentation is common to other bacterial enterocolitis, including those caused by *Yersinia* [119].

### 7.2. Extraintestinal Manifestations

Extraintestinal complications of *Campylobacter* infection are uncommon and typically occur following bacteremia. Among these, osteomyelitis is reported, especially involving the vertebral column, and in immunocompromised patients; however, cases have also been reported in otherwise healthy individuals [120,121,122].

Meningitis, although uncommon, can occur, particularly in vulnerable populations such as newborns and individuals with underlying neurological conditions [123,124].

Cardiac involvement is an extremely rare complication; cases of myocarditis have been primarily described in young men without predisposing factors and with a generally good prognosis [125]. Co-infection with other viruses is suspected to play a role in the development of cardiac complications [126].

### 7.3. Late Complications

#### 7.3.1. Guillan–Barré Syndrome, Miller Fisher Syndrome

Guillain–Barré syndrome (GBS) is a rapidly progressing, immune-mediated polyradiculoneuropathy often triggered by a preceding infection, most commonly involving the respiratory or the gastrointestinal tract [127]. Depending on the anatomical structures affected and the resulting clinical manifestations, various subtypes can be identified: the predominant subtype is AIDP (acute inflammatory demyelinating polyneuropathy), with AMAN (acute motor axonal neuropathy), AMSAN (acute motor-sensitive axonal neuropathy), and Miller Fisher syndrome (MFS), a triad of ophthalmoplegia, ataxia, and areflexia, representing other distinct clinical presentations.

Approximately 75% of GBS cases are preceded by a clinically evident infection; among bacteria, *Campylobacter* and *Mycoplasma* are the main ones [128]. The first evidence of an association between *Campylobacter* infection and GBS dates to Rhodes in 1982 [129]. Based on International Guillain–Barré syndrome Outcome Study (IGOS) consortium data reported by Leonhard et al. in 2022, approximately 30% of GBS patients have a history of *Campylobacter* infection. Notably, *Campylobacter* would be more strongly associated with the development of the AMAN subtype, less with AIDP [130]. These results corroborate the findings of a systematic review by Poropatich et al. in 2010, which highlighted a 31% prevalence of campylobacteriosis preceding GBS [131].

The onset of neurological symptoms can occur between 10 days and 3 weeks after the onset of diarrhea [132]. Molecular mimicry between surface oligo-saccharide antigens and myelin gangliosides is the leading pathogenic mechanism. Beyond this, other pathways contributing to axonal damage have been described. These include complement-mediated damage through the membrane attack complex (MAC), direct effects of C3a and C5a, TNF-alpha-driven macrophage activation, and oxidative damage caused by hydroxyl radicals [133]. Other complement activation pathways, including the mannose-binding lectin (MBL) pathway, and interactions involving oligo-saccharides and SIGLEC-1 on antigen-presenting cells (APC) may also contribute to axonal injury [134].

According to Heikema, different serotypes, such as HS:19 and HS:41, would have increased pathogenicity due to alterations in surface polysaccharides [135].

Indeed, in MFS, possible molecular mimicry predisposes to the formation of anti-ganglioside GM1, GD1a, and GQ1b IgG antibodies, the latter being present in the myelin of cranial nerves [136]. The HS:2 and HS:4 complex serotypes would be those most associated with MFS [137].

Regarding unilateral oculomotor nerve palsy, *Campylobacter* is known to be one of the main predisposing factors for the formation of anti-ganglioside GQ1b antibodies [138].

#### 7.3.2. Reactive Arthritis

Reactive arthritis, formerly known as Reiter’s syndrome, is a type of seronegative spondyloarthropathy often triggered by gastrointestinal infections [139]. Among the most frequently involved bacteria are *Campylobacter jejuni*, *Escherichia coli* (*O157*:*H7*), *Salmonella enteriditis*, *Shigella flexneri* and *dysenteriae*, and *Yersinia enterocolitica* [140]. Molecular mimicry is also likely involved in this condition, with arthritis usually developing within 4 weeks of infection. Symptoms can be monoarticular, oligoarticular, or polyarticular; the most affected joints are the large joints of the lower limb, such as the knees or ankles, although small joints may also be involved. The duration of symptoms is variable, with Pope et al. reporting a mean duration of 6 months in their systematic review [141].

## 8. Pediatric Population

The 0–4 age group has been consistently identified in multiple studies as having the highest incidence of campylobacteriosis [142,143]. Children can be exposed to environmental contaminants during outdoor activities, such as playing in parks or gardens. Inadequate hand hygiene practices and exposure to environmental contaminants are likely major contributing factors; suburban settings present numerous potential environmental reservoirs for infectious agents. Parks, soil, and bodies of untreated water, particularly those contaminated with fecal matter from reservoir hosts, are notable examples [14,144]. The studies by Colles et al. underscore the role of urban wildlife as a contributing factor to environmental contamination with *Campylobacter* [145,146]. Beyond ingestion, children can be exposed to foodborne pathogens through direct contact with contaminated food sources, such as raw or undercooked meat. The relative risk of enteropathogenic bacterial infections is elevated by a factor of at least two among individuals who consume raw meat or unpasteurized milk [147]. As noted by Fullerton et al., contaminated hands of caregivers can be a significant source of infection transmission [148]. The study by Pyra et al. underscores the importance of caregiver education in preventing child infection: knowledge of transmission routes, facilitated by educational interventions, leads to enhanced hygiene practices [149]. Furthermore, in children older than six months, repeated and close contact with domestic animals is a notable risk factor [148]. According to Diriba et al. and Lengerh et al., contact with domestic animals increases the risk of acquiring *Campylobacter* by up to 3.2 times [150,151].

Children under five years of age who are colonized with enteric pathogens are at increased risk of malnutrition [152,153]. *Campylobacter* infections in children are typically mild and self-limiting; however, in infants, severe complications, including mortality, have been reported [118]. The clinical picture of enteritis may include severe abdominal pain and a high degree of fever and lead to severe dehydration. Approximately 50% of patients with enteritis present with bloody stools [154]. Younger children tend to shed the bacteria for longer periods, with durations ranging from 3 days to several months. Additionally, although rare, symptoms may have a relapsing course, recurring after an initial resolution.

## 9. Management: From Diagnosis to Treatment

A diagnosis of *Campylobacter* infection is suspected based on the patient’s history and clinical findings. Microbiological confirmation requires the isolation of the bacteria through culture or the detection of its DNA using molecular techniques.

Typically, *Campylobacter* infection presents as enterocolitis with diarrhea, often associated with blood and mucus in the stools. A history of consuming raw or undercooked meat (especially poultry), unpasteurized milk, or untreated water; swimming in untreated water; or travel to low-resource countries increases the suspicion of campylobacteriosis. Less common but possible is direct contact with domestic animals.

Laboratory findings commonly reveal elevated inflammatory markers, including C-reactive protein, and neutrophilia. However, leukopenia has also been observed in some cases [155,156].

Abdominal ultrasound often demonstrates evidence of inflammatory bowel disease, characterized by thickened bowel walls with preserved layers, particularly involving the colon, and reactive mesenteric lymphadenopathy. This exam is a valuable tool in distinguishing between acute ileocecitis caused by *Campylobacter* and acute appendicitis [157].

Microbiological diagnosis is essential for confirmation and includes culture test, performed on stool samples, blood in case of bacteremia, or histological specimens; molecular NAATs (nucleic acid amplification tests) offer high sensitivity and are commonly used [158].

*Campylobacter* infection is usually self-limiting and mild. According to Ternhag et al., meta-analysis, while antibiotics may slightly shorten the duration of symptoms by about 12–24 h, the overall clinical benefit is limited [159]. Furthermore, there is no evidence that antibiotics increase the risk of shedding the pathogen or causing relapse.

Regarding the use of specific antimicrobial therapy, international guidelines agree in recommending it for severe intestinal infections, characterized by persistent dysentery or signs of severe and systemic infection. Host factors, including malnutrition, immunodeficiency, and malignancy, can also influence the decision to treat.

Azithromycin is widely proposed as the preferred initial antibiotic treatment considering the increasing resistance to FQs, especially those from Asia.

According to the 2024 Yellow Book recommendations for travelers’ diarrhea, published by the Centers for Disease Control and Prevention (CDC), antibiotic therapy is not recommended for mild forms of diarrhea. For moderate forms, antibiotic use is possible, while it is recommended for severe manifestations. Azithromycin is the preferred antibiotic over FQs and Rifaximin. Pediatric patients are at increased risk of dehydration from severe diarrhea and should be closely monitored. Rifaximin is not approved for use in children under 12 years of age and should only be considered as a second-line option for older children [160]. The World Gastroenterology Organisation (WGO) recommends considering antibiotic therapy for *Campylobacter* dysentery. Treatment is strongly advised for persistent dysentery, especially in patients at high risk of dehydration or with underlying conditions like lymphoproliferative diseases, chronic illnesses, and severe malnutrition. Azithromycin remains the first-line treatment, with FQs as a second option. In children, trimethoprim-sulfamethoxazole (TMP-SMX) is indicated as an alternative [161]. The Infectious Diseases Society of America (IDSA) agrees that antibiotic therapy can be appropriate for severe cases. Azithromycin is the preferred treatment, while FQs are considered second-line options and should generally be avoided in Asian countries. TMP-SMX is a third-line alternative [162]. The primary therapeutic recommendations are presented in Table 2

## 10. Antimicrobial Resistance

A marked escalation in antibiotic resistance among *Campylobacter* strains was observed as early as the 1990s [163]. The commensal relationship between bacteria and livestock animals raised for meat production and human infections leads to a sustained exposure of the bacteria to antimicrobial agents. The continual exposure to antibiotics has resulted in the selective survival of resistant strains that have emerged over time. Macrolides are the first-line treatment for human campylobacteriosis, with fluoroquinolones, aminoglycosides, and tetracyclines as alternative options. In veterinary practice, florfenicol is commonly used [118]. The Centers for Disease Control and Prevention (CDC) has identified antibiotic resistance in *Campylobacter* as a ‘significant public health threat’, as detailed in its 2019 report. According to the report, a significant proportion (approximately 29%) of *Campylobacter* isolates, including both *C. jejuni* and *C. coli*, in the United States are resistant to FQs or macrolides [164].

*Campylobacter* is considered multidrug-resistant (MDR) when it exhibits resistance to three or more distinct classes of antibiotics [165]. Multidrug resistance most commonly occurs against FQs, macrolides, trimethoprim-sulfamethoxazole, tetracyclines, and florfenicol [144,166]. A significantly higher proportion of multidrug-resistant isolates, exceeding 60%, is reported in Italy and Portugal compared to other European countries [167,168,169]. Complementing the CDC’s data, Dias et al. reported that 35.7% of *Campylobacter* isolates in Brazil exhibited multidrug resistance, highlighting a significant public health concern in the Americas [170]. The most concerning data come from Asia, with MDR rates reaching 87.3% in Korea and exceeding 98% in Thailand [171,172]. Among continents, Oceania, with Australia as a notable example, demonstrates a lower incidence of resistant strains [173].

Mechanisms underlying antibiotic resistance encompass reduced permeability of the bacterial cell wall, increased efflux pump activity, alterations in drug target sites, and enzymatic inactivation of the antibiotic. These mechanisms often synergize, conferring resistance to multiple drug classes.

In addition to these mechanisms, *Campylobacter* can acquire resistance determinants through horizontal gene transfer (HGT), particularly through transformation and conjugation [174]. Scott et al. in 2007 proposed an additional role for bacteriophages in this process, a topic of continued investigation [175,176,177].

### 10.1. Fluorquinolone Resistance

Fluoroquinolones (FQ) are the most commonly used quinolones and exhibit excellent activity against Gram-negative bacteria. Once inside the cell, they lead to DNA strand breakage by binding to DNA gyrase and topoisomerase IV. DNA gyrase consists of two subunits, GyrA and GyrB, whereas topoisomerase IV comprises ParcC and ParcE subunits; the former pair is found in *Campylobacter*, whereas ParcC and E are not [178,179]. *C. coli* and *C. jejuni* can develop mutations in GyrA, specifically within the quinolone-resistance determining region (QRDR), while GyrB remains unaffected [180]. Specifically, the Thr86Ile mutation confers a significant increase in FQ resistance (MIC ≥ 16 μg/mL), whereas other mutations result in lower levels of resistance (MIC = 1–8 μg/mL) [178,181].

Another highly effective mechanism of both intrinsic and acquired resistance to FQs is mediated by the CmeABC efflux pump; this molecule is the primary efflux pump in *Campylobacter* and is highly conserved within the genus. CmeABC reduces intracellular drug accumulation [182,183,184,185]. Yao et al. have shown that a CmeB variant, named RE-CmeABC, where the variable part is in the CmeB subunit, increases the efficiency of the efflux pump in the presence of GyrA mutations (MIC ≥ 256 μg/mL) [186]. These data emphasize the importance of this system in the survival of strains with GyrA mutations [187]. Furthermore, this efflux pump also contributes to resistance against bacteriocins, peptides produced by bacteria [188]. Other pump systems, such as CmeDEF and CmeG, appear to play a role in *Campylobacter* resistance, although to a lesser extent [189,190].

Rising rates of quinolone resistance in *Campylobacter* have been documented since the 2000s. Zhou et al. demonstrated a substantial increase in ciprofloxacin resistance among *C. jejuni* isolates in China over a 17-year period, from 50% to 93% [191]. Similar findings were reported by Wang and Li, revealing an alarmingly high prevalence of FQ resistance in both swine and poultry isolates, approaching 100% [192,193]. A global trend of increasing FQ resistance in animal isolates has been observed, with studies from China, as well as numerous Asian, European, and American countries, supporting this finding [194,195,196,197,198,199,200,201]. Another highly effective mechanism of both intrinsic and acquired resistance to FQs is mediated by the CmeABC efflux pump; this molecule is the primary efflux pump in *Campylobacter* and is highly conserved within the genus. CmeABC reduces intracellular drug accumulation.

### 10.2. Macrolide Resistance

*Campylobacter* can develop macrolide resistance through two primary mechanisms: target site alterations and antibiotic efflux. Target site alterations can be achieved either through single-point mutations or through enzyme-mediated methylation. Mutations at positions 2074 and 2075 of the domain 5 result in the inability of the antibiotic molecule to bind to 23S rRNA [202,203]. Mutation of one of the three copies of 23S rRNA results in lower resistance, while modifications of all three copies result in higher resistance (MIC > 128 μg/mL) [204,205,206]. Modifications to ribosomal proteins L4 and L22 represent an alternative mechanism of macrolide resistance, resulting in intermediate levels of resistance [207]. An important rRNA methylating enzyme found in both *C. jejuni* and *C. coli* is Erm(B); gene *erm(B)* can be located on both the chromosome and plasmids [208,209]. Its presence confers high levels of macrolide resistance; moreover, it is associated with multidrug resistance genomic islands (MDRGIs), which can be transferred by transformation or plasmid-mediated conjugation [208]. Similarly to its role in FQ resistance, the CmeABC efflux pump plays a major role in macrolide resistance [184,210].

### 10.3. Beta-Lactam Resistance

Beta-lactam resistance is mediated by two primary mechanisms: enzymatic inactivation by beta-lactamases and active efflux by MDR pumps. OXA-61 (Cj0299) is the sole beta-lactamase identified in *Campylobacter* to date, though others may be present. Single promoter mutations can dramatically increase OXA-61 expression, up to 256-fold [211,212]. The CmeABC efflux pump also contributes to resistance to beta-lactams [213].

### 10.4. Tetracycline Resistance

*Campylobacter* resists tetracyclines by employing both the CmeABC efflux pump and the protective protein Tet(O). Tet(O) is an elongation factor-like protein that confers resistance to the protein synthesis inhibitor tetracycline by promoting the release of the drug from its inhibitory site on the ribosome [214].

### 10.5. Aminoglycoside Resistance

Aminoglycoside resistance can occur through different mechanisms: efflux pumps, 16S rRNA mediation, mutations in the rRNA binding site, active swarming, and enzymatic modification of the antibiotic molecule. Modifications introduced by phosphotransferases I, III, IV, and VII, through phosphorylation of hydroxyl groups, confer resistance to aminoglycosides such as gentamicin, kanamycin, and neomycin [215]. Additionally, acetyltransferases may contribute to resistance phenotype [216,217].

### 10.6. Florfenicol Resistance

Florfenicol is a fluorinated derivative of thiamphenicol and has been widely used in veterinary medicine since the mid-1990s. The primary mechanisms of resistance include modification of the 23S rRNA target through Cfr(c) methyltransferases or mutations, increased efflux via pumps, and enzymatic activation through chloramphenicol acetyltransferases [165].

## 11. Conclusions and Future Prospects

Although the infection is often asymptomatic in poultry, human campylobacteriosis is a significant public health concern, being one of the most prevalent bacterial gastroenteritis worldwide. Even though the infection is self-limiting and has a low mortality rate, the healthcare costs are extremely significant due to the high incidence. The control measures can be divided into two main phases: pre-harvest and post-harvest interventions. Primary biosecurity measures include improving environmental hygiene to minimize *Campylobacter* exposure through the cleaning and replacement of soils and vegetation on the farm, reducing the mixing of different species, and limiting contact with other possible reservoirs such as wild animals. Implementing rigorous biosafety and biosecurity protocols for farm workers is essential [218,219]. Despite numerous trials, food additives, including prebiotics and probiotics, have proven ineffective in significantly reducing colonization and spread of the germ [220,221,222]. Additionally, there is a critical need to develop alternative antibiotic therapies and a vaccine to address the growing problem of multidrug resistance in avian populations. Post-harvest procedures focus on the thorough sanitation of the entire production chain, including farms, transportation, and end products such as carcasses and egg shells.

The development of a vaccine aims to reduce the intestinal colonization of poultry. Although numerous vaccines have been proposed in recent years, none have shown sufficient efficacy to provide a definitive solution. Even before the year 2000, the whole cell vaccine (WCV), consisting of killed or attenuated cells and administered orally, was proposed; however, it demonstrated minimal efficacy [223]. Subunit vaccines targeting *Campylobacter* virulence factors, including flagellin and CjA attenuated-*Salmonella* vectored, have been investigated but have failed to provide effective protection [224]. Recent trials have focused on non-particle-based vaccines delivered orally, via the intranasal route, or in ovo; these vaccines utilize encapsulation within nanoparticles containing C. jejuni lysates or recombinant plasmids expressing the flagellin (*flgA*) gene [225]. Vaccines incorporating CjA and CjD delivered by Gram-positive Enhancer Matrix (GEM) particles have shown encouraging results [226].

The convergence of factors, including the widespread prevalence of *Campylobacter*, increased poultry production, global travel, and the emergence of antibiotic resistance, necessitates a comprehensive approach to develop and implement effective containment strategies. A comprehensive strategy combining improved hygiene practices, biosafety measures, the synergistic use of food additives and pre- and probiotics, vaccine development, and enhanced public health awareness is essential for effective *Campylobacter* control.

## Figures and Tables

**Table 2:** Summary of international recommendations for the antibiotic treatment of *Campylobacter* jejuni/coli infection.

Source	Clinical Manifestations	Treatment	First-Line Antibiotic	Second-Line Antibiotics
**CDC Yellow Book** [160] (2024)	mild	not recommended	none	none
	moderate	possible	azithromycin	fluoroquinolones, rifaximin (>12 years)
	severe	recommended		
**World Gastroenterology Organisation** [161] (2012)	dysentery	consider	azithromycin	fluoroquinolones, trimethoprim-sulfamethoxazole (children)
	persistent dysentery	recommended		
	patients at high risk of dehydration	recommended		
**Infectious Diseases Society of America** [162] (2017)	immunocompromised patient	consider	azithromycin	fluoroquinolones (should be avoided in Asian countries), trimethoprim-sulfamethoxazole
	severe	recommended		
	infants < 3 months	recommended		
	sepsis-like	recommended		

## Data Availability

No new data were created or analyzed in this study. Data sharing is not applicable to this article.
